# Understanding motorcycle rider behavior and the related traffic contexts in Indonesia using naturalistic driving study

**DOI:** 10.1016/j.heliyon.2025.e42494

**Published:** 2025-02-10

**Authors:** Winda Halim, Maya Arlini Puspasari, Ridwan Aji Budi Prasetyo, Yusuf Ardiansyah, Listiani Nurul Huda, Idham Halid Lahay, Lovely Lady, Fatin Saffanah Didin, Manik Mahachandra, Hardianto Iridiastadi

**Affiliations:** a Faculty of Industrial Technology, Institut Teknologi Bandung, Indonesia; b Bachelor Program in Industrial Engineering, Faculty of Smart Technology and Engineering, Universitas Kristen Maranatha, Bandung, Indonesia; c Department of Industrial Engineering, Faculty of Engineering, Universitas Indonesia, Indonesia; d Department of Psychology, Faculty of Social and Political Sciences, Brawijaya University, Indonesia; e Department of Industrial Engineering, Faculty of Engineering, Universitas Sumatera Utara, Indonesia; f Department of Industrial Engineering, Faculty of Engineering, Universitas Negeri Gorontalo, Indonesia; g Industrial Engineering Department, University of Sultan Ageng Tirtayasa, Indonesia; h Department of Industrial Engineering, Institut Teknologi Sumatera, Indonesia; i Industrial Engineering Department, Engineering Faculty, Diponegoro University, Indonesia

**Keywords:** Roadway crash, Behavior, Motorcycle, Naturalistic driving study, Safety critical events

## Abstract

Motorcycle crash fatalities remain a significant concern in Indonesia. Understanding the traffic events leading to such crashes is crucial for designing effective mitigation strategies. This preliminary study aimed to identify and analyze safety-critical events (SCEs) experienced by motorcycle riders. Using a naturalistic approach, 79 participants from six cities were observed while riding their motorcycles during daily routines, covering a total of 1036 km. A camera mounted on the rear-view mirror captured their riding behavior and traffic situations. Trained analysts conducted interviews with participants and reviewed video footage to identify and quantify SCEs. The study found no significant differences in the number of SCEs across cities but revealed notable differences in their causes. While rider behavior was a key factor, a substantial proportion of SCEs were attributed to the actions of other road users, such as near crashes caused by overtaking between opposing traffic. These findings highlight the need for comprehensive mitigation strategies, including rider education, improved road design, and better traffic signage.

## 1 Introduction

Motorcycles are the most widely used mode of transportation in Indonesia due to their practicality and affordability. The number of registered motorcycles has grown exponentially since 2019 [1], placing Indonesia at the top of the list among Southeast Asian countries with the highest motorcycle ownership rates. Currently, over 40 % of the population owns a motorcycle, accounting for 85 % of motorized vehicles on the road [2]. However, this surge in motorcycle ownership has led to a significant increase in roadway accidents. Motorcycle crashes account for nearly 73 % of all traffic accidents [3], resulting in economic losses of approximately 250 billion rupiah in 2021. These accidents are also associated with 25,000 fatalities and over 10,000 injuries annually. The actual figures could be even higher, as road traffic incidents are often underreported in developing countries [4].

Human error is the leading contributing factor to accidents [5]. These errors can be classified into unsafe behaviors, traffic rule violations, and inadequate enforcement of regulations [6,7]. In the United States, for example, human error is the primary cause of 57 % of accidents and a contributing factor in 90 % of cases [8]. Similarly, in Malaysia, 71–74 % of serious motorcycle accidents are linked to rider behaviors, such as failing to use turn signals, stopping beyond the stop line, or not paying attention to other road users [9,10]. Among these factors, distracted driving has been identified as the leading cause of fatal motorcycle accidents in Malaysia [11]. Rider age also plays a crucial role in road safety [12,13]. Inexperienced riders often struggle to recognize potential hazards and respond appropriately [14]. Furthermore, they are frequently reluctant to acknowledge the need for behavioral changes that could improve their driving skills and overall safety.

One approach to understanding the causes of crashes involves conducting focus group discussions (FGDs) with motorcyclists aimed at gathering their perspectives on riding behavior, risk factors, and related strategies or technical issues [15]. While FGDs provide valuable initial insights and recommendations, they rely on subjective opinions and require validation through more objective methods. Another approach is crash data analysis, which involves collecting information from sources such as police reports, hospitals, and other authorities. Researchers use this data to develop crash scenarios and analyze motorcyclist behavior [16,17]. However, this method may not fully capture real-world rider behavior, as decisions made during crashes are influenced by multiple factors that may not be reflected in the data.

The most common and accessible method for measuring rider behavior is the use of questionnaires. These have been employed to model near-miss incidents [18] and identify factors contributing to motorcycle accidents [19]. One of the most widely adopted is the Motorcycle Rider Behavior Questionnaire (MRBQ) [20], which has been adapted for use in various countries, including Thailand [21], Vietnam [22], Iran (Persian version) [23], and Australia [24]. However, a key limitation of this approach is the subjective nature of self-reported data. A more advanced and experimental method involves the use of motorcycle simulators to observe rider behavior. These simulators recreate realistic crash scenarios, enabling researchers to capture objective responses in controlled environments [25]. While this approach provides valuable insights, the behavior observed in simulations may not fully reflect natural responses in real-world situations.

Research on motorcyclist behavior in Indonesia and its relationship to traffic accidents has been widely conducted, with most existing studies relying on subjective approaches. These studies identify and discuss various factors contributing to accidents, such as underage drivers [26], non-compliance with helmet use [27], traffic flow dynamics, road conditions, the proportion of motorcycles on the road, and the presence of medians and road shoulders [28]. Other factors include the use of cell phones while driving [29], violations of traffic regulations [30], and gender differences in accident involvement [31]. However, subjective or self-reported methods may not accurately capture the actual behavior of drivers. To address this limitation, more objective and factual approaches, such as direct field studies using the Naturalistic Driving Study (NDS) method, are necessary.

NDS approach is invaluable for understanding rider behavior [15], with findings that can inform the development of effective training programs and regulatory changes. NDS aims to observe everyday riding behavior while accounting for vehicle conditions and surrounding traffic environments [32]. The first large-scale NDS study was conducted on 100 cars [33], but research specifically focused on motorcycles remains limited. Notable large-scale motorcycle NDS projects include 2BeSafe in Europe [34] and MSF 100 in the United States [35].

Previous NDS research has primarily aimed to understand rider behavior and its relationship with conditions that lead to safety-critical events (SCEs) [36,37]. These studies have examined factors such as human-related aspects [[37], [38], [39]], vehicle-specific characteristics [[40], [41], [42]], and environmental conditions [43]. Insights from NDS data have been utilized to identify and analyze vulnerable situations for motorcyclists [44], improve understanding of lane splitting [36], and evaluate how new riders respond to risky situations [37]. These findings have been used in the development of training programs for new riders and the enhancement of traffic regulations.

NDS-based research can be broadly classified into three categories: (i) baseline, normative, or exposure research, which focuses on observing rider behaviors and performance; (ii) critical accident and near-crash research, which aims to identify characteristics and investigate factors contributing to accidents; and (iii) system-focused research, which examines the interaction between riders and vehicle systems [45].

The methods used for data collection and processing vary significantly depending on the study's objectives. Common approaches include asking riders to maintain a logbook of their riding activities, document risky events, and record riding situations, which are later confirmed through interviews with participants [36,37,44]. Other procedures involve conducting a pilot study to define and measure the subjects, followed by a quasi-experimental design in the main study, and culminating in a naturalistic riding study (NRS). The final results are often presented using g-g diagrams to analyze and detect rider profiles [46].

Evaluating traffic problems can be challenging, as many factors—both internal and environmental—can influence driving activities. Rider-related factors may include, but are not limited to, riding skills, motivation for traveling, personal norms, and other individual characteristics. On the other hand, environmental factors may encompass weather conditions, traffic density, the behavior of other drivers, and the enforcement of traffic regulations, among others. A comprehensive study is essential to understand the behavioral aspects of riders and how they respond to external factors.

Understanding motorcycle accidents in Indonesia presents unique challenges. Due to their affordability compared to other motorized vehicles, motorcycles are frequently modified for various purposes, such as e-hailing, transporting large or heavy goods, towing extended carts, or carrying more than two passengers. It is common for riders to navigate through very tight and narrow spaces, zigzag between larger vehicles, or disregard traffic signs and regulations. These practices create significant risks, not only for the riders and their passengers but also for other road users. Additionally, the diversity of roads in Indonesia—varying in type, design, quality, and how they are used by other road users—further complicates driving. What might be considered dangerous or unusual driving behavior in other countries, such as overtaking, lane changes, and aggressive maneuvers, is often seen as the norm in Indonesia. However, these distinct driving behaviors, along with the unique traffic conditions, have been rarely studied.

The objective of this study was to examine potential traffic conflicts (safety-critical events) using a naturalistic approach. This method provides a direct way to capture motorcyclists' riding patterns, their interactions with other road users, and prevailing traffic conditions. The research offers valuable insights into driver responses to various road complexities, such as navigating traffic jams, empty roads, steep or winding terrains, and areas with high pedestrian activity, like street markets. By analyzing driver behavior, the influence of other road users, and interactions with the environment, NDS-based studies can inform interventions to promote safer driving habits. The findings of this study are expected to serve as a foundation for developing mitigation strategies, including education, road and signage redesign, and regulatory enforcement.

## 2 Methods

### 2.1 Data collection

**Fig. 1:**
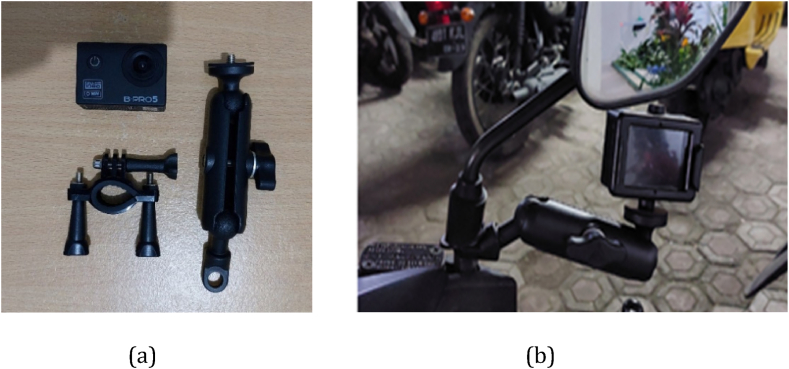
Research instrumentation (a) camera Brica Pro 5 and mount; (b) installation on the rear-view mirror.

The Naturalistic Driving Study (NDS) data collection method was employed, adapted from the literature review of Aupetit et al. [37]. It utilized field observation and a method of recording driving activities through the use of a camera. In this study, only one camera was used for recording, positioned at the front of the motorcycle, facing the roadway. To ensure clear imagery and accurate recording for data collection, the camera was mounted on the participant's motorcycle's rear-view mirror, allowing it to be easily removed and reinstalled on each participant's bike. Efforts were made to position the camera centrally to capture a more precise viewing angle. The camera used was an action camera with a 170-degree field of view. The type of camera and mount is shown in Fig. 1(a), while its placement is illustrated in Fig. 1(b).

The travel route for this research was not predetermined. Participants were instructed to activate their cameras while traveling from one location to another during their daily routines, such as from home to office or vice versa. They were asked to ride for a full day, using their own motorbikes to commute to work, school, or make leisurely trips to various places. Most participants were familiar with the routes, as they travelled them daily. Data recording conditions were mostly conducted in sunny weather to ensure accurate recordings.

**Fig. 2:**
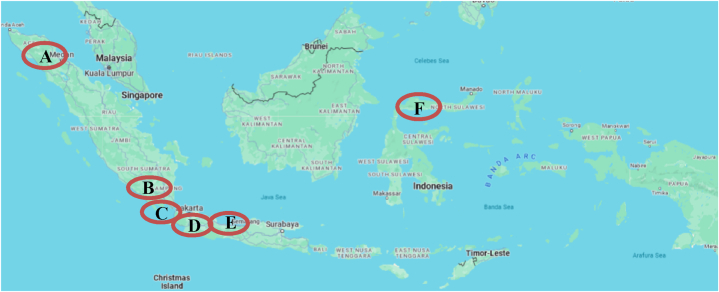
A map of Indonesia and cities where data were obtained.

Data collection was conducted in six cities across three major islands in Indonesia, as shown in Fig. 2. The cities include three on Java Island, two on Sumatra Island, and one on Sulawesi Island. Five of these cities are provincial capitals, characterized as major cities, while one is an industrial city, situated near industrial centers and ports. Bandung, the capital of West Java, is a major city with uphill and downhill roads due to its highland location, experiencing high traffic density. Cilegon City, an industrial city in northern Java, and Semarang, the capital of Central Java, follow similar urban patterns. Medan, the capital of North Sumatra, has dense roads but less disciplined road users, with motorbikes and motorized rickshaws dominating public transport. Bandar Lampung, the capital of Lampung province, serves as the gateway to Sumatra Island. Gorontalo, on Sulawesi Island, is a more remote city with diverse geographical conditions. Fig. 2 illustrates the location of each city.

The cities selected for this study were chosen purposively to represent diverse cultures and socioeconomic statuses in Indonesia. For instance, Medan (A) is a major economic hub and the third-largest city in Indonesia, whereas Gorontalo (F) is a smaller city in a remote area, home to significant ethnic groups like the Batak and Gorontalo. Bandar Lampung (B) has undergone cultural fusion due to transmigration from Java, while Cilegon city is a prominent industrial center. Bandung (C) and Semarang (E) are typical provincial capitals in Java, with strong service sectors and mixed cultures. The diverse geographical context of the study ensures a comprehensive dataset, reducing bias and increasing generalizability, particularly by avoiding over-representation of Java Island, where motorcycle registration numbers surpass other islands.

A total of six analysts were involved in this study. Their roles included interviewing the participants for the purpose of validating SCEs. The analysts had been trained in determining what constituted an SCE. The determination of SCEs in each city was based on the research of Aupetit et al. [37] and was developed according to the conditions of drivers in each city. The data collection process was standardized across all cities, including procedures, camera type, placement, and recording methods. Contributors in each city subjectively assessed video footage to identify SCEs, guided by a predefined list of SCEs established during a pilot project. Subjectivity was minimized as each analyst was already briefed and trained during a technical meeting prior to data collection process.

### 2.2 Data processing

To analyze risky behaviors of motorcyclists, driving records from participants were collected and processed. Data visualizations are used to identify key trends, including the number and proportion of risky situations, as well as the characteristics of each situation. These data could be used to in the development of intervention recommendations. Data reduction techniques (done by trained analysts) were applied to quantify risky situations and categorize them based on causal factors. This study considered three primary causal factors: (i) motorcyclist behavioral factors, based on Aupetit et al.'s research [37]; (ii) behavioral factors of other road users [37]; and (iii) traffic conditions [47].

Data reduction involved extracting essential data from naturalistic video studies to identify risk situations, situational contexts, and characteristics. In this study, adjustments were made to accommodate limited stages. The data reduction process yielded situations experienced by participants, factors causing these situations, categories of risky situations, and their characteristics. The extracted risk situation contexts and characteristics included journey type, travel habits, road type, infrastructure, interactions with other road users, weather, and road conditions [37]. These aligned with the NDS study's data collection area and participant demographics.

### 2.3 Statistical method

In NDS research, accidents are rarely recorded, making safety-critical events (SCEs) a valuable substitute approach [49]. SCEs can be perceived as hazardous or non-hazardous, depending on the driver's perspective, and are often triggered by road users' driving behavior in specific situations [37,48]. However, SCEs are challenging to define, infrequent, and difficult to predict, requiring extensive NDS data to ensure none are overlooked [50].

SCEs are categorized into four types [48].

1. Crashes: contact between a motor vehicle and an object in the traffic environment.
2. Near-crashes: situations requiring excessive driving maneuvers to avoid a collision.
3. Crash-relevant conflicts: excessive maneuvers to prevent a crash, without resulting in critical conditions.
4. Unintentional lane deviations: vehicles crossing dividing lines without posing immediate danger.

Note that this study did not identify any SCEs in the crash category.

The collected trip records were then reduced to determine the number of occurrences of SCEs caused by the driver, other road users, and traffic conditions. The identification of SCEs and their number of occurrences were done by each observer subjectively in each city. The number of similar SCE events was then calculated for each city and cause. The data obtained will be processed using several statistical methods, including the Kruskal-Wallis Test to see the differences between SCEs in each city (cities A-F) and between each cause (drivers, other road users, and traffic conditions). In addition, a correlation test was conducted to see the correlation between the number of SCEs in each city and the driving distance and time. The coefficient of determination was also calculated to see the percentage influence of distance and driving time on the SCEs obtained.

## 3 Results

### 3.1 Demographic data

This research was conducted in six cities (A, B, C, D, E, and F) located on three major islands in Indonesia. City E, situated on Java Island, boasts the largest area, spanning 373.8 km^2^. In contrast, City A has the highest number of motorbikes, with 113,324 per 100,000 population, indicating a higher number of motorbikes than population. When comparing motorbike numbers to area, City F exhibits the highest density. Refer to Table 1 for detailed data on each city.

**Table 1:** Number Motorcycle per 100.000 population.

City	Area (km^2^)	Number of motorcycles per 100.000 population	Number of motorcycles per km^2^
A	265,1	113324	427
B	197,2	89796	455
C	162,5	82657	509
D	167,3	90638	542
E	373,8	94161	252
F	79,59	58730	738

The participants of this study were 79 motorcyclists with a total distance travelled of 1036.06 km and a total duration of 39.69 h. Details related to this information can be seen in Table 2. Most participants were young riders aged 20–30 years old with less than 10 years of riding experience, except in city D where the average age was more than 30 years old with more than 10 years of riding experience. Most participants had more than 5 years of riding experience, implying that participants could be considered as having sufficient riding experience.

**Table 2:** Demographic data.

Demographic Data		Age (years)	Distance (km)	Duration (minutes)	Experiences (years)
A (n = 14)	Mean	22.00	6.01	14.19	2.64
	Std. dev	2.66	4.07	12.05	1.34
B (n = 10)	Mean	24.70	12.29	22.97	5.80
	Std. dev	4.22	3.13	6.52	3.22
C (n = 15)	Mean	23.33	8.88	21.26	4.27
	Std. dev	3.60	3.64	11.17	1.98
D (n = 10)	Mean	33.30	9.82	29.42	16.70
	Std. dev	15.73	5.11	13.68	15.41
E (n = 10)	Mean	24.70	12.36	22.35	6.30
	Std. dev	9.60	2.53	5.82	5.91
F (n = 20)	Mean	24.25	10.87	19.83	4.80
	Std. dev	2.36	1.18	3.78	2.31

### 3.2 Safety critical event (SCE) in each city

**Fig. 3:**
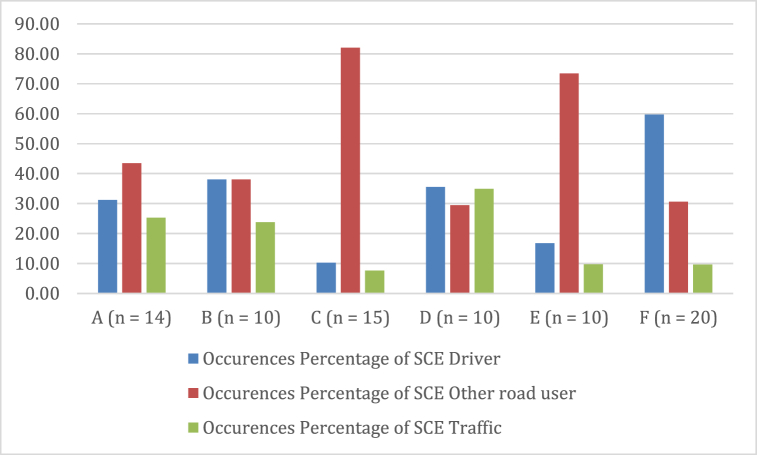
Percentages of SCE in each city and the corresponding causes.

When utilizing Naturalistic Driving Studies (NDS), crash situations are rarely observed, prompting the use of Surrogate Crash Events (SCEs) to identify potential risks. However, the accuracy of SCE identification can be rather subjective and influenced by the analyst's experience and knowledge. This, in turn, affects the type and number of SCEs that can be identified. Video observations in each city enabled the determination and counting of SCEs, with results presented in Fig. 3. SCEs were categorized into three causal factors: driver own behavior, behavior of other road users, or traffic conditions. In cities A, C, and E, other drivers/road users were the primary factor, while in cities B and D, the driver was the main contributor. Notably, in city B, drivers and other road users contributed equally to SCEs.

For the standardized indicator of the number of situations per kilometer, all the cities observed scored below one per kilometer. However, city C scores above one per kilometer for risky situations caused by other road users. This means that in at least 1 km of driving, participants in city C encounter one risky situation caused by other road users. The ratio can be seen in Table 3.

**Table 3:** Ratio of cause of the situation based on who caused it per kilometer.

City	Self/Km	Other Road Users/Km	Traffic Conditions/Km
A	0,385	0,588	0,260
B	0,102	0,122	0,061
C	0,207	1654	0,154
D	0,316	0,263	0,311
E	0,154	0,672	0,089
F	0,170	0,087	0,028

Risky situations in observed cities could be attributed to various factors, including trip purpose, travel habits, road type, infrastructure, interactions with other road users, weather, and road conditions. Personal trips posed a higher risk, while daily commuting was the most dangerous based on trip style. In urban areas, highways are the most common location for hazardous incidents, despite also occurring on other infrastructure. In observed cities, motorcyclists are the most frequently involved with other road users, likely due to the high number of motorbike riders in Indonesia. Data collection during favourable weather conditions may not fully capture hazardous situations that occur in other weather conditions. Road conditions significantly contribute to hazardous situations, and manual data calculation was used to identify risky situations. The characteristics of trips made by participants can be found in Table 4.

**Table 4:** Characteristics of trips.

Context	Characteristics	City					
		A	B	C	D	E	F
Trip purpose	Home to workplace (round-trip)	68	11	0	87	9	0
	Personal Trips	86	31	298	51	56	46
	Home to school or campus (round-trip)	83	0	42	28	48	16
Trip style	Daily commuting (Usual)	185	36	155	115	72	12
	Leisure	52	6	185	51	41	50
Types of Road	City/municipal	227	37	197	134	46	62
	Rural	6	0	8	29	19	0
	Neighbourhood/driveway	4	0	135	3	6	0
	Fast Highway	0	0	0	0	42	0
	Sumatra interstate	0	3	0	0	0	0
	District	0	2	0	0	0	0
Road infrastructure	Curve	0	3	2	13	12	1
	Intersection	54	8	34	20	21	3
	Highway	176	28	299	127	71	58
	Separators	3	0	0	3	2	0
	Roundabout	0	1	2	1	2	0
	Entrance/exit lane	0	0	3	0	3	0
	Acceleration lane	0	0	0	0	2	0
	Flyover	3	2	0	1	0	0
	Bridge	1	0	0	1	0	0
Involvement	Motorcycle	130	22	299	85	86	52
	Car	85	14	32	43	13	6
	Others	12	3	0	26	3	1
	Pedestrian	4	1	5	6	4	2
	Truck	6	2	1	5	6	1
	Bus	0	0	3	1	1	0
Weather	Clear	219	42	332	153	108	62
	Rainy	18	0	0	13	5	0
	Windy	0	0	8	0	0	0
Road conditions	Wet	33	0	56	14	5	0
	Normal	202	40	280	145	108	62
	Damaged	2	0	4	7	0	0
	Potholed	0	2	0	0	0	0

The SCE that occurs most frequently in each city can be seen in Table 5. According to the table, City C, an industrial city, has the highest SCE compared to other cities. In contrast, City B has the smallest SCE value. The most common SCE is "Common traffic violations undertaken by participants or other road users," which includes examples such as passing through a red light. This SCE indicates that there is still a high number of violations occurring due to participants and other road users.

**Table 5:** Most frequent SCE in each city.

City	SCE	Occurrences	Percentage
A	Another road user from the opposite direction drove on the wrong side/lane (using participant's lane).	45	18,99 %
B	Participant experienced a near-crash with another road user who performed sudden lane-changing in front of the participant.	8	19 %
C	Common traffic violations undertaken by participants or other road users.	213	62.65 %
D	Participant experienced a near-crash with another road user during overtaking vehicles.	49	29,52 %
E	Common traffic violations undertaken by participants or other road users.	38	33.63 %
F	Participant overtook from the right-side when there was an oncoming vehicle.	15	24,20 %

### 3.3 Statistical analysis

The number of events obtained from the six cities was differentiated based on the cause by the observer. To determine whether there were significant differences between the six cities (City A-F) and the three causes (driver, other road user, and traffic), tests were conducted. The Kruskal-Wallis test was used to assess whether the six cities had significantly different values of SCE occurrence. The results showed a p-value >0.05, indicating no significant difference in SCE percentage between cities. This may be due to varying traffic characteristics not affecting SCE frequency. Table 6, Table 7 display the Kruskal-Wallis test results for the six cities and three causes. Notably, the p-value <0.05 for the three causes suggests significant differences in SCE causes, leading to the rejection of the null hypothesis.

**Table 6:** Kruskal Wallis test for six cities.

Method	DF	H-Value	P-Value
Not adjusted for ties	5	0.98	0.964
Adjusted for ties	5	0.98	0.964

**Table 7:** Kruskal Wallis test for three causes.

Method	DF	H-Value	P-Value
Not adjusted for ties	2	7.56	0.023
Adjusted for ties	2	7.57	0.023

A correlation test was conducted between the total number of occurrences of SCEs in each city, total travel distance, and total driving time. A low negative correlation was obtained between the number of SCEs and total driving distance (−0.3658) and between the number of SCEs and total driving time (−0.0067). This indicated that the amount of SCE was not related to the distance and time travelled, so the farther or longer people drive did not necessarily result in increased SCE. When testing using the coefficient of determination (R^2^), it was shown that only 13.38 % of traveling distance affects the number of SCEs and only 0.0045 % of traveling time affected the number of SCEs. Based on these results, it can be said that there are probably other factors besides distance and duration that affected the number of SCEs.

## 4 Discussion

This preliminary first Indonesian study explored potential traffic conflicts (termed "critical events") using a naturalistic approach. Key findings indicate that no significant differences in SCEs were found across six cities (five provincial capitals and one industrial city). However, two cities exhibited a substantial role of other road users in creating traffic conflicts. A situation that exemplified unique traffic conflict is simultaneous overtaking by two motorcycles in opposite directions creating hazardous situations. This study highlights the complexity of traffic conflicts in Indonesia, emphasizing the need for comprehensive solutions addressing both rider behavior and other road users' actions.

### 4.1 Uniqueness of SCEs in Indonesia

The naturalistic approach is widely used in research; however, the characteristics of SCEs can differ between developing and industrialized nations. In the present study, such differences were in fact shown between cities with different backgrounds. This study found distinct results across cities, reflecting variations in motorcyclist demographics, environmental factors, and road conditions. When conducting research using NDS, it is uncommon to be able to capture the occurrence of crashes. Therefore, a surrogate approach to crash events is adopted, namely Safety Critical Events (SCE) [49]. SCE can be triggered by driving behaviour of road users in a driving situation [48]. Understanding the characteristics of SCE that commonly occur can be used to design appropriate policies or mitigations to reduce the number of incidents. There are differences in safety critical events between developing and developed countries. This is due to differences in road user habits, available infrastructure, and applicable traffic regulations. Indonesia, as a developing country, has its own unique characteristics, for example, lane changing [36], which is illegal and dangerous in other countries, is considered normal and common in Indonesia. At present, Indonesia does not have dedicated motorcycle lanes. Motorcycles are the predominant vehicles on the road and share space with other vehicles. In some areas of major cities, there are designated waiting spaces for motorcycles located immediately before a pedestrian crossing. A waiting space area will be used when the traffic light turns red, and motorcycles will gather in that area. In some major cities, motorcycles are also used to pull rickshaws, commonly known as motorized rickshaws or powered three-wheeler, therefore it can carry more than one passenger.

The results from city C and E showed that motorcyclists committed more common violations, such as riding on sidewalks, running traffic lights, or not wearing riding gear such as a helmet. The percentage of common violations in these two cities is relatively high, reaching 62.65 % in City C and 33.64 % in City E. Interestingly, in Cities D and F, the most frequent SCE involves drivers nearly colliding with other road users while overtaking, accounting for 29.52 % and 24.20 % of cases, respectively. This is likely due to drivers' lack of caution when overtaking and the prevalence of narrow, two-lane roads in these urban areas. Cities D and F have high traffic density, as indicated by the number of motorcycles per kilometer. In City A, the most common violation involves road users from the opposite direction entering the wrong lane, accounting for 18.9 % of total violations. City A is known for lower traffic discipline among motorists, which may explain the prevalence of this violation. Finally, in City B, the most common SCE involves a driver nearly colliding with another road user who suddenly changes lanes in front of them. This accounts for 19 % of total occurrences. This could be attributed to human error, as the driver may have failed to notice the lane change of the vehicle ahead [24].

### 4.2 Factors influencing SCEs

Based on the data, human factors—particularly the actions of other drivers and motorcyclists—are the primary causes of road crashes, compared to traffic and environmental factors. This highlights the significant role of human factors in driving [51,52] and underscores that riders are the most aware of the hazard levels in driving situations [53]. The Kruskall-Wallis test also revealed significant differences in the causes of SCEs. Traffic conditions in Indonesia are different from other developing countries. In Indonesia, motorcycles account for up to 85 % of vehicles, yet motorcycle-specific regulations remain inadequate [2]. Transportation safety regulations only cover helmet use and passenger limits, with no provisions for road rules or dedicated motorcycle lanes. Many motorcyclists lack awareness of the importance of following regulations, and some intentionally commit violations [30]. This is evident from the high number of intentional violations by motorcyclists. Additionally, enforcement of laws against unsafe motorcyclist behavior is weak.

### 4.3 Implications and limitation

The theoretical implication of this study is to understand the characteristics of driving behavior in six cities in Indonesia. The findings indicate that most violations result from interactions with other drivers, such as near collisions during overtaking, wrong-way driving by opposing traffic, or sudden lane changes by other road users. These findings highlight the need for policies that promote motorcyclist awareness and safer driving behaviors. This can be achieved by enhancing rider training programs and implementing stricter licensing procedures to ensure competency. Stronger law enforcement is also needed to improve motorcyclist discipline. Motorcycle safety cannot be achieved individually but requires a systematic and collective effort.

The limitation of this research is that it was only conducted in several cities in Indonesia so the data obtained are limited and observations using only video camera footage without the use of other instruments. Moreover, the identification of SCE is done by different trained analysts, so the level of accuracy in identifying SCE could also differ. In-vehicle movement sensor or riders’ physiological data were not collected along the naturalistic driving study, since the focus of this study were behavior situation of the riders and their surroundings. However, to validate their behavior, short interviews were also conducted after each driving session to note any incidents occurred. For future research, the number of samples and number of days to collecting data needs to be increased to improve the accuracy of the results. Other measurements such as sensors and physiological instruments could also be applied in the future research.

## 5 Conclusions

This study aimed to understand how Safety Critical Events (SCEs) occur, focusing on rider behavior, other road users' actions, and the unique characteristics of traffic situations. The Naturalistic Driving Study (NDS) method was applied to develop interventions that promote safer rider behavior. The results showed that the most frequent SCEs in six Indonesian cities involved motorcyclists’ violations linked to other road users' lack of awareness when overtaking, failing to notice lane changes ahead, or encountering vehicles moving in dangerous or inappropriate directions.

Other common violations included disregarding traffic signals, running red lights, and not wearing a helmet. These findings align with previous research indicating that near misses during lane changes in dense traffic are among the most frequent violations. A key practical implication is the need for improved education and road design, including better markings. Strategies to mitigate lane-changing violations should include technologies such as collision avoidance systems in motor vehicles. Stronger enforcement of traffic regulations is also needed, particularly for lane changing, riding on sidewalks, running red lights, and not wearing helmets [54].

## CRediT authorship contribution statement

**Winda Halim:** Writing – original draft, Methodology, Conceptualization. **Maya Arlini Puspasari:** Investigation. **Ridwan Aji Budi Prasetyo:** Methodology, Investigation. **Yusuf Ardiansyah:** Investigation. **Listiani Nurul Huda:** Investigation. **Idham Halid Lahay:** Investigation. **Lovely Lady:** Investigation. **Fatin Saffanah Didin:** Investigation. **Manik Mahachandra:** Investigation. **Hardianto Iridiastadi:** Writing – original draft, Conceptualization.

## Informed consent statement

Informed consent was obtained from all subjects involved in the study.

## Data availability statement

Data will be made available upon request from the corresponding author.

## Funding

This study was made possible using research funding provided by the Institut Teknologi Bandung and Universitas Kristen Maranatha.

## Declaration of competing interest

The authors declare that they have no known competing financial interests or personal relationships that could have appeared to influence the work reported in this paper.
